# Interlaboratory comparison of *Pseudomonas aeruginosa* phage susceptibility testing

**DOI:** 10.1128/jcm.00614-23

**Published:** 2023-11-14

**Authors:** Krupa Parmar, Lauren Komarow, Damon W. Ellison, Andrey A. Filippov, Mikeljon P. Nikolich, Joseph R. Fackler, Martin Lee, Anjna Nair, Priyesh Agrawal, Pranita D. Tamma, Maria Souli, Scott R. Evans, Kerryl E. Greenwood-Quaintance, Scott A. Cunningham, Robin Patel, for the Antibacterial Resistance Leadership Group

**Affiliations:** 1 Division of Clinical Microbiology, Department of Laboratory Medicine and Pathology, Mayo Clinic, Rochester, Minnesota, USA; 2 Biostatistics Center, George Washington University, Rockville, Maryland, USA; 3 Wound Infections Department, Bacterial Diseases Branch, Walter Reed Army Institute of Research, Silver Spring, Maryland, USA; 4 Adaptive Phage Therapeutics Inc., Gaithersburg, Maryland, USA; 5 Division of Pediatric Infectious Diseases, Department of Pediatrics, Johns Hopkins University School of Medicine, Baltimore, Maryland, USA; 6 Duke Clinical Research Institute, Durham, North Carolina, USA; 7 Department of Biostatistics and Bioinformatics, Milken Institute School of Public Health, George Washington University, Washington, D.C., USA; 8 Division of Public Health, Infectious Diseases, and Occupational Medicine, Department of Medicine, Mayo Clinic, Rochester, Minnesota, USA; Johns Hopkins University, Baltimore, Maryland, USA

**Keywords:** phage susceptibility testing, liquid assay, plaque assay, *Pseudomonas aeruginosa*

## Abstract

Standardized approaches to phage susceptibility testing (PST) are essential to inform selection of phages for study in patients with bacterial infections. There is no reference standard for assessing bacterial susceptibility to phage. We compared agreement between PST performed at three centers: two centers using a liquid assay standardized between the sites with the third, a plaque assay. Four *Pseudomonas aeruginosa* phages: PaWRA01ø11 (EPa11), PaWRA01ø39 (EPa39), PaWRA02ø83 (EPa83), PaWRA02ø87 (EPa87), and a cocktail of all four phages were tested against 145 *P*. *aeruginosa* isolates. Comparisons were made within measurements at the two sites performing the liquid assay and between these two sites. Agreement was assessed based on coverage probability (CP_8_), total deviation index, concordance correlation coefficient (CCC), measurement accuracy, and precision. For the liquid assay, there was satisfactory agreement among triplicate measurements made on different days at site 1, and high agreement based on accuracy and precision between duplicate measurements made on the same run at site 2. There was fair accuracy between measurements of the two sites performing the liquid assay, with CCCs below 0.6 for all phages tested. When compared to the plaque assay (performed once at site 3), there was less agreement between results of the liquid and plaque assays than between the two sites performing the liquid assay. Similar findings to the larger group were noted in the subset of 46 *P*. *aeruginosa* isolates from cystic fibrosis. Results of this study suggest that reproducibility of PST methods needs further development.

## INTRODUCTION

The antibiotic resistance crisis has led to renewed interest in phage therapy, leveraging natural predators of bacteria to control bacterial infections. Phages are highly specific toward their bacterial hosts, a strength that limits off-target effects on normal microbiota while simultaneously rendering it necessary to determine that a phage selected for therapeutic use is indeed active against pathogenic bacterial isolates (1). Selecting an ideal phage candidate for therapeutic application requires testing individual phages against each patient’s bacterial isolate using phage susceptibility testing (PST). In contrast to conventional antibiotic susceptibility testing, there are no standardized PST methods and no validated interpretive criteria. Ideally, interpretive criteria or breakpoints for PST would be determined based on clinical outcomes, but since evaluation of clinical outcomes is in the process of being determined, such data are not currently available. In the meantime, standardized and reproducible approaches for PST are essential to optimize phage clinical trials and make individualized decisions on compassionate therapy.

Two primary PST methods are used, the double agar overlay method that assesses plaque formation as the indicator of phage activity and liquid assays that assess bacterial growth or metabolism in the presence of phage (2). For the former, plaque formation is observed following rounds of infection, lysis, and release of progeny phages on the same spot, resulting in zones of clearance. Visibility and size of plaques depend on the phage latent period, burst size, diffusion rate in soft agar, and bacterial growth (3). Efficiency of plating (EOP) quantifies relative efficiencies with which different cells are infected by viruses and support viral replication by assessing the ratio of plaque counts to numbers of virions in the inoculum (4). Liquid assays, based on delayed rises in bacterial optical density measured at 600 nm (OD_600_) or bacterial respiration as measured using redox dyes because of phage infection, for example, may be performed at relatively high throughput such as using 96-well plates.

Definitions of ideal experimental conditions, such as standardized phage and bacterial concentrations [and ratios—multiplicity of infection (MOI)], bacterial growth phases, media compositions, incubation temperatures, durations of assessment, quality control metrics, and result interpretation for PST, are lacking. Recently, the PhageScore (5) and Phage Virulence Index (6) were described to assess phage activity in liquid assays to select phages for clinical applications. The PhageScore provides a quantification of lytic activity based on area under the curve for evaluation and comparison of a phage’s infectivity against different bacterial hosts. The PhageScore is calculated for a specific MOI and varies for different MOIs of the same phage. On the other hand, the Phage Virulence Index is a standardized quantitative method to assess phage virulence based on three parameters: the Virulence Index, quantifying virulence of a phage against its specific host; the local virulence, assessing killing potential at a given MOI; and MV_50_, the MOI at which the phage achieves 50% of its maximum theoretical virulence. The Biolog liquid assay based on respiration of bacteria uses “hold time”—the time delay in reaching log-phase metabolic activity when a bacterium is infected with a phage (7).

Examples of parameters that may limit phage activity and therefore affect assessment of phage activity during PST include (1) poor adsorption to bacterial surfaces, (2) absence or suboptimal structure of phage receptors, (3) blockage of entry of phage DNA into bacteria by superinfection exclusion, (4) infection prevention by restriction-modification or CRISPRs, or (5) abortive infection where cell death occurs before progenies are produced (8). Beyond these, other novel bacterial resistance mechanism systems have been explored that stop phage predation (9). Since phage evolution is high, phages lack conserved genomic regions like 16S ribosomal RNA genes in bacteria, phage databases are limited and contain undefined hypothetical proteins, and bacterial resistance mechanisms to phage predation are diverse, it can be tricky to predict phage susceptibility using molecular methodologies. In measuring susceptibility to a cocktail of phages, potential competition for host receptors and blockage of superinfection exclusion by another phage may limit susceptibility. Given that there is no reference standard for assessing PST, parameters that need to be assessed for individual assays include within-day, between-day, and between-site agreements of results generated using the same and different methods, ideally using many bacterial isolates and multiple phages.

**Fig 1:**
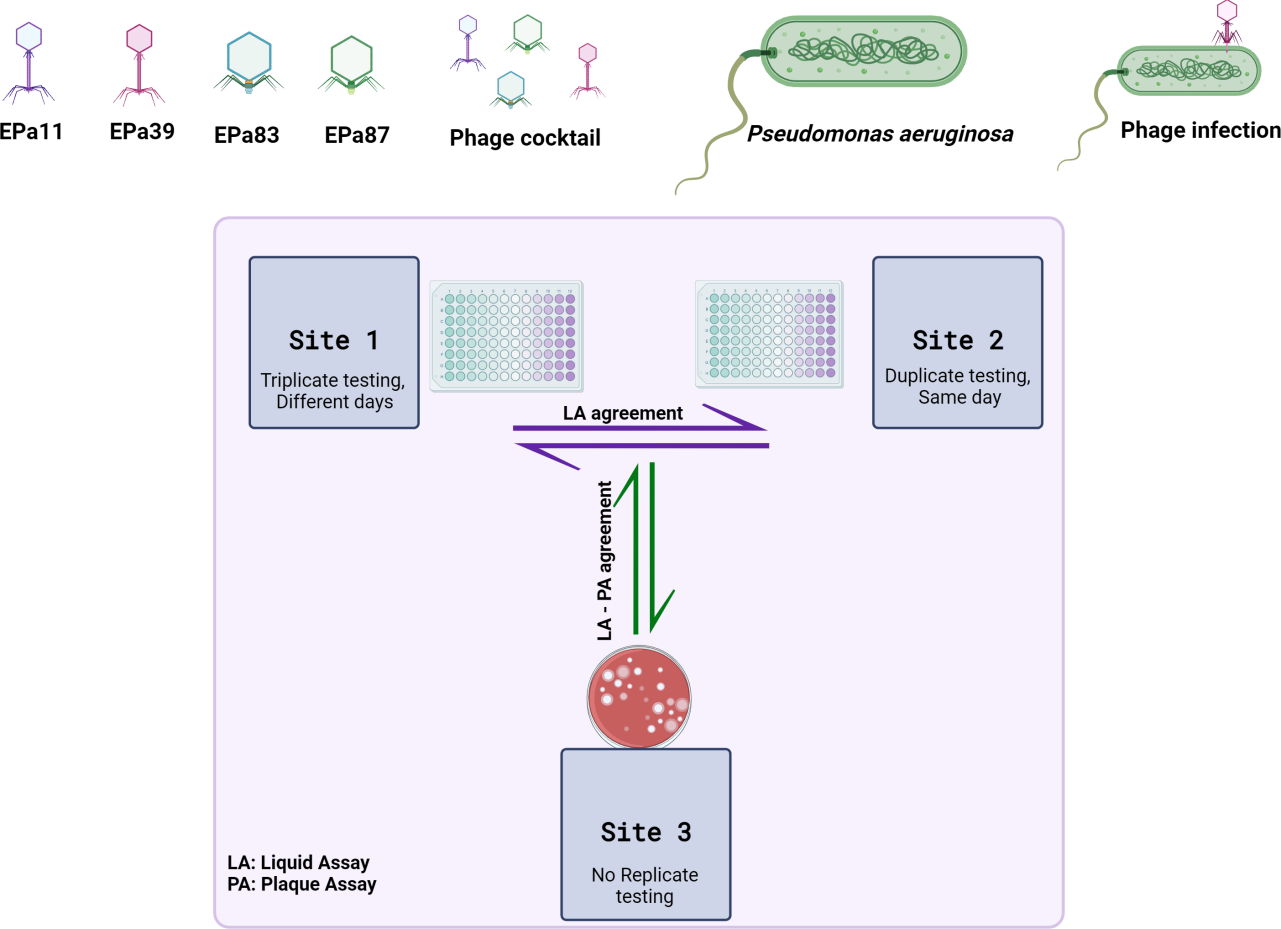
Graphical abstract (created with BioRender.com).

Herein, results of a liquid assay PST method performed using the Biolog OmniLog in two laboratories (site 1, where testing was performed on three different days, and site 2, where testing was performed in duplicate on single runs) were compared with each other and to results of a double-layer plaque assay performed once at site 3 (Fig. 1), using four phages and a cocktail mixture of all four against 145 *Pseudomonas aeruginosa* isolates. Agreement was assessed comparing three repeated liquid assay measurements at site 1, two repeated liquid assay measurements at site 2, median liquid assay measurements from sites 1 and 2, liquid assay measurements performed at sites 1 and 2, and plaque assays performed at site 3.

## MATERIALS AND METHODS

PST was performed in three laboratories: the Antibiotic Resistance Leadership Group Laboratory Center, Mayo Clinic (site 1); Adaptive Phage Therapeutics, Inc. (APT, site 2); and the Walter Reed Army Institute of Research (site 3). Four phages individually (Table 1) and a cocktail of the four were evaluated against 145 *P*. *aeruginosa* respiratory isolates including a subset of 46 isolates from cystic fibrosis patients. The phages are being evaluated in a phase 1b/ 2 clinical trial in cystic fibrosis patients (NCT05453578) (10). The phage cocktail was prepared at a ratio of 1:1:1:1 of the four phages and tested at the same total concentration as individual phages. Two PST methods were assessed: (1) a double-agar overlay plaque assay for EOP (11) and (2) a liquid assay modification of a previously described Biolog OmniLog® method (7, 12).

**TABLE 1:** Phages studied^*a*^

Phage	Genome size (bp)	Family	Genus	Bacterial host
PaWRA01ø11 (EPa11)	66800	*Myoviridae*	*Pbunavirus*	PaWRA01
PaWRA01ø39 (EPa39)	66708	*Myoviridae*	*Pbunavirus*	PaWRA01
PaWRA02ø83 (EPa83)	45439	*Podoviridae*	*Bruynoghevirus*	PaWRA02
PaWRA02ø87 (EPa87)	44893	*Podoviridae*	*Bruynoghevirus*	PaWRA02

^*a*^ The phages are being evaluated in a phase 1b/ 2 clinical trial (NCT05453578).

For the plaque assay, the phages were serially diluted, and 2 µL of each dilution (10^−1^ to 10^−8^) was plated using an eight-channel micropipette on the 5-mL soft agar overlay infused with 100 µL of overnight grown *P. aeruginosa* isolates. Plates were incubated at 37°C overnight, and zones of clearance and isolated plaques observed on the bacterial lawn. Results were classified as active (i.e., phage-infected bacteria), based on plaque formation, and inactive, when plaques were not observed.

For the liquid assay, bacterial metabolism was assessed by color change associated with a tetrazolium dye indicator over 48 hours in a 96-well microtiter plate assay. Bacterial isolates were grown in trypticase soy broth (TSB, MilliporeSigma) on a shaker incubator at 37°C. Bacterial inoculum was adjusted to an Optical density measured at 600nm (OD_600_)of 0.085–0.115 and stored on ice up to 2 hours before plate setup. The medium used for testing was a fresh mix of 1% (vol/vol) Biolog tetrazolium Dye D (50 µL) (Biolog, Inc.) in (5-mL) TSB, sterilized with a Steriflip 0.22-µm vacuum filter (MilliporeSigma) and stored at 2°C to 8°C in the dark until use. Ninety-six-well Biolog plates were filled with 80-µL tetrazolium dye/TSB along with 10-µL test phage (final concentration 1 × 10^6^ PFU/mL) and 10-µL test bacteria (final concentration 1 × 10^5^ CFU/mL). Phages with their bacterial host (EPa11 and EPa39 with bacterial host PaWRA01; and EPa83 and EPa87 with bacterial host PaWRA02; Table 1) positive controls, along with media (100 µL), bacterial hosts (10 µL in a 90-µL medium), and phage stocks (10 µL in a 90-µL medium), were used in each plate for quality control. Plates were inserted in the Biolog OmniLog® instrument at 37°C, and readings made every 15 minutes over 48 hours. Data were analyzed using the OmniLog Data Analysis Software (version 1.7, Biolog, Inc.), and hold times calculated using PhageSelect™ web-based software (APT). Background noise was normalized using PhageSelect’s algorithm to calculate log-phase growth time inflections. Phage activity was assessed as the inability of the isolate to establish a logarithmic growth phase relative to the uninfected control in the presence of phage, with the length of time the phage prevented bacterial growth from entering the log phase measured over 48 hours. Hold times (in hours) were determined by subtracting inflection times of phage and bacterial combinations from inflection times of bacterial growth controls ranging from 0 to 48 hours. Hold times of 4 hours or higher were considered representative of active phage based on a study which demonstrated that phages exhibiting hold times of less than 4 hours resulted in variable activity when spotting on agar overlay (APT, personal communication). Accordingly, hold times less than 4 hours were considered to suggest lack of phage activity in a recent study (7). Here, hold times of 8 hours were also evaluated as a more stringent cutpoint.

Sites 1 and 2 performed liquid assays which resulted in quantitative results in terms of hold times. At site 1, testing was performed in triplicate on three different days, whereas at site 2, testing was performed in duplicate on single runs. Site 3 performed single-plaque assays which resulted in qualitative (categorical) results of active/inactive phage. Reproducibility of liquid assays was compared pairwise directly and separately among tests at each site and between results of the two sites. Results from the liquid method from both sites were also compared to those of the plaque assay. Intra-site results for liquid assay runs reported for sites 1 and 2 were analyzed. Inter-site results for liquid assay runs were also analyzed. Intra- and inter-site results were separately analyzed for the cystic fibrosis isolated subgroup and non-cystic fibrosis subgroup. An inter-site analysis comparing liquid assay with plaque assay results was also performed.

Intra-site and inter-site comparisons for the liquid assay were made between the maximum and minimum of two (site 2) or three measures (site 1) for each isolate and each phage type, and statistical properties were evaluated for maximum difference between measurements. If there was disagreement (active/inactive) between any two sets of measures (1 and 2, 2 and 3, or 1 and 3), comparison between the maximum and minimum values would disagree. Agreement of runs was assessed based on (i) accuracy and precision of measurements, (ii) total deviation index (TDI_0.9_), (iii) coverage probability π (CP_8_), and (iv) concordance correlation coefficient (CCC) (13 – 15). Accuracy was defined as whether there was disagreement between the two distributions (mean and variance) of the measures (13); precision measured variation between the two samples. In this study, since there is no actual true value of accuracy, accuracy was defined as the value which was the “same” for liquid assays at both sites. If one of the values did not match another, it was considered inaccurate. Considering that the actual plaque-forming units and hold times of each phage-bacterium combination are unknown, the true value/accuracy is unavailable for comparison. Here, measurement of accuracy is closeness of a value among the hold times of two measurements; hence, the formula of accuracy measures both the mean and standard error to quantify the shift in distributions; TDI_0.9_ is the value of the difference between matched pairs such that 90% of the matched pairs have a difference less than this value. CP_8_ is the proportion of matched pairs with a difference of less than 8. CCC takes a value of 1, indicating perfect agreement, a value of −1, indicating perfect disagreement, and a value of 0, indicating no agreement. CCC is the product of the accuracy and precision coefficients.

For inter-site comparisons between the liquid assay and plaque assay (between sites 1 and 3 and between sites 2 and 3), positive percent agreement (PPA) and negative percent agreement (NPA) were plotted as functions of a range of thresholds for pairwise comparisons, with the plaque assay as the reference.

## RESULTS

At site 1, triplicate measurements of the liquid assays of five phage preparations (four phages and one cocktail mixture) were tested for agreement. Pairwise comparisons among three measurements within site 1 are shown using scatter plots in Fig. S1, with color coding based on plaque assays at site 3. Scatterplots show comparisons of first and second, second and third, and first and third measurements at site 1. Measurements are quantified on the diagonal line of each plot, with correlation between the two measurements considered linear. Results of comparisons are shown as CCC values, precision coefficients, accuracy coefficients, and TDI_0.9_ and CP_8_ values in Table 2. For phage EPa83, the CCC value of 0.83 (95% CI 0.79–0.86) indicates strong excellent agreement between the maximum and minimum values, with high precision [0.87, 95% confidence interval (CI) 0.85–0.90] and accuracy coefficient (0.95, 95% CI 0.93–0.96) values. Phage EPa39 had the smallest TDI_0.9_ value (9.63, 95% CI 9.03–10.3); 90% of the paired (maximum and minimum of three measures) observations had a difference of less than 9.63. Phage EPa39 showed the least overall activity, likely influencing it having the smallest TDI_0.9_ value and high CP_8_. However, accuracy and precision measures were lower at 0.78 (95% CI 0.74–0.83) and 0.66 (95% CI 0.58–0.72), respectively. Results are visualized in scatter plots in Fig. S1D through F; measures lie diagonally for phage EPa83, while for phage EPa39, there is no linear relationship. Phage EPa83 had strong agreement but was mostly inactive (Fig. S1G through I). For phages EPa11 and EPa87, although the CCCs were low at 0.66 (95% CI 0.60–0.71) and 0.60 (95% CI 0.54–0.66), respectively, precision and accuracy values were acceptable. The phage cocktail also exhibited a low CCC but satisfactory precision (0.80, 95% CI 0.76–0.84) and accuracy (0.86, 95% CI 0.83–0.89) as visualized in the scatter plots (Fig. S1M through O).

**TABLE 2:** Agreement between maximum and minimum of three measurements at site 1 (*N* = 145, TDI_0.9_, CP_8_)^*a*^

Phage	CCC (95% CI^*b*^)	Precision (95% CI)	Accuracy (95% CI)	TDI_0.9_ (95% CI)	CP_8_ (95% CI)
EPa11	0.66 (0.60–0.71)	0.76 (0.71–0.81)	0.86 (0.82–0.89)	16.6 (15.4–17.9)	0.64 (0.61–0.68)
EPa39	0.52 (0.45–0.59)	0.66 (0.59–0.72)	0.79 (0.74–0.83)	9.63 (9.0–10.3)	0.93 (0.92–0.94)
EPa83	0.83 (0.79–0.86)	0.87 (0.85–0.90)	0.95 (0.93–0.96)	12.0 (11.0–13.1)	0.75 (0.72–0.79)
EPa87	0.60 (0.54–0.66)	0.72 (0.66–0.78)	0.83 (0.79–0.87)	13.9 (12.9–14.9)	0.76 (0.74–0.79)
Cocktail	0.69 (0.64–0.74)	0.80 (0.76–0.84)	0.86 (0.83–0.89)	17.9 (16.6–19.3)	0.55 (0.51–0.58)

^*a*^ Concordance correlation coefficient (CCC) is the product of the accuracy and precision coefficients where accuracy is used to decide whether there is disagreement between two distributions (mean and variance) of the measures and precision measures variation between two samples. A CCC value of 1 indicates perfect agreement; a value of −1 indicates perfect disagreement, and a value of 0 indicates no agreement. Total deviation index (TDI_0.9_) is the difference between matched pairs such that 90% of matched pairs have a difference less than this value. CP_8_ is the proportion of matched pairs with a difference of less than 8.

^*b*^ CI, confidence interval.

**Fig 2:**
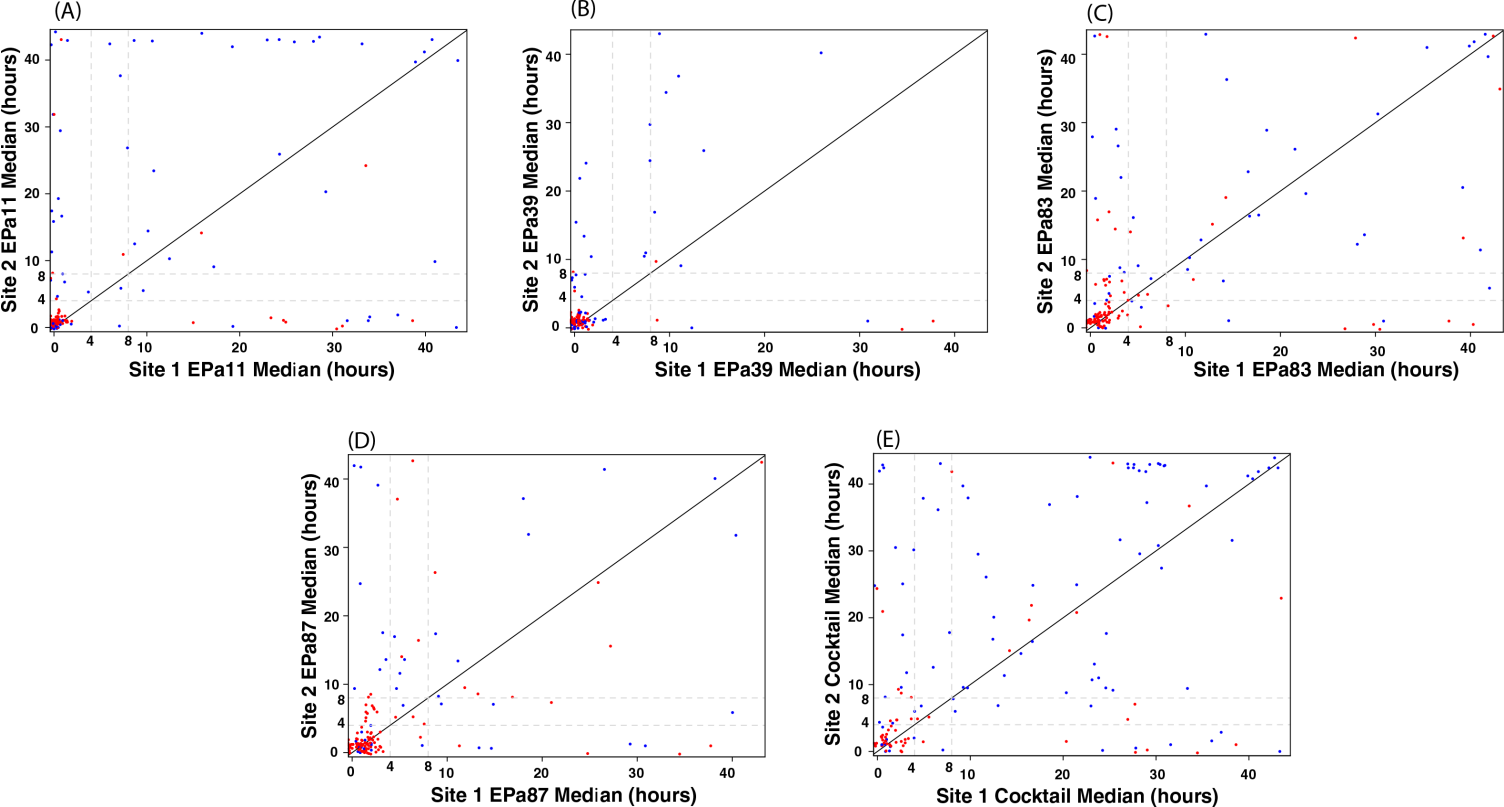
Scatterplot comparing agreement among median values of three measurements at site one and two at site 2, using a liquid assay cutpoint of four hours. Color coding is based on plaque assays at site 3: red - inactive; blue - active. (**A**) phage EPa11, (**B**) phage EPA39, (**C**) phage EPa83, (**D**) phage EPa87, and (**E**) phage cocktail.

The five phage preparations (four phages and the cocktail) were also tested for agreement of the two repeated measurements at site 2 (Table 3). Scatterplots comparing the two parallel run measurements are shown in Fig. S2. All phage preparations had CCCs greater than 0.836, where the precision coefficients (0.839–0.911) were close to the CCC and the accuracy coefficient was larger than 0.99. CP of differences no larger than eight for all five phage preparations was greater than 0.7, implying that the probability of paired observations having a difference of less than 8 hours was at least 70% for all phage preparations tested. Accuracy of measurements was fairly high, while precision was intermediary, implying that the measurements were close to each other but with a lot of variation. Overall, duplicate measurements at site 2 showed a high level of agreement and accuracy as compared to triplicate measurements at site 1, likely because there was variability from the third run at site 1, and because runs were tested on different days, with different bacterial and phage inocula at site 1. Site 2 used the same bacterial inoculum and phage inoculum for each run, which likely minimize variability that might be attributed to uncertainty around inoculum size and stage of bacterial growth. A comparison of agreement (Fig. 2) among median values of three measurements at site 1 and median values of two measurements at site 2 is shown in Table 4. The CCCs for all phage preparations were close to 0.5; TDI_0.9_ values were greater than 10, and the CP_8_ was highest for phage EPa39; although the accuracy was fair, the precision was very low. As seen from the scatterplots shown in Fig. 2, measurements of all phages at both the sites were in low agreement.

**TABLE 3:** Agreement between two measurements at site 2 (*N* = 145, TDI_0.9_, CP_8_)^*a*^

Phage	CCC (95% CI)	Precision (95% CI)	Accuracy (95% CI)	TDI_0.9_ (95% CI)	CP_8_ (95% CI)
EPa11	0.91 (0.88–0.93)	0.91 (0.89–0.93)	0.99 (0.98–0.99)	10.7 (9.71–11.8)	0.78 (0.73–0.82)
EPa39	0.84 (0.79–0.87)	0.84 (0.79–0.88)	0.99 (0.97–0.99)	7.72 (7.00–8.51)	0.91 (0.87–0.94)
EPa83	0.86 (0.82–0.89)	0.86 (0.82–0.89)	1.00 (0.95–1.00)	11.1 (10.1–12.2)	0.76 (0.71–0.81)
EPa87	0.90 (0.87–0.92)	0.90 (0.87–0.92)	1.00 (0.98–1.00)	8.02 (7.28–8.84)	0.90 (0.86–0.93)
Cocktail	0.91 (0.88–0.93)	0.91 (0.88–0.93)	1.00 (0.97–1.00)	11.8 (10.7–13.0)	0.73 (0.68–0.78)

^*a*^ Concordance correlation coefficient (CCC) is the product of the accuracy and precision coefficients where accuracy is used to decide whether there is disagreement between two distributions (mean and variance) of the measures and precision measures variation between two samples. A CCC value of 1 indicates perfect agreement, a value of −1 indicates perfect disagreement, and a value of 0 indicates no agreement. TDI_0.9_ is the difference between matched pairs such that 90% of matched pairs have a difference less than this value. CP_8_ is the proportion of matched pairs with a difference of less than 8.

**TABLE 4:** Agreement between median measurements at sites 1 and 2 (*N* = 145, TDI_0.9_, CP_8_)^*a*^

Phage	CCC (95% CI)	Precision (95% CI)	Accuracy (95% CI)	TDI_0.9_ (95% CI)	CP_8_ (95% CI)
EPa11	0.44 (0.32–0.54)	0.45 (0.34–0.56)	0.96 (0.90–0.98)	23.8 (21.6–26.3)	0.42 (0.38–0.45)
EPa39	0.44 (0.35–0.52)	0.55 (0.45–0.64)	0.80 (0.74–0.85)	11.4 (10.3–12.5)	0.75 (0.70–0.79)
EPa83	0.56 (0.46–0.65)	0.56 (0.46–0.65)	0.99 (0.94–1.00)	19.0 (17.2–20.9)	0.51 (0.47–0.55)
EPa87	0.46 (0.35–0.56)	0.48 (0.36–0.58)	0.97 (0.92–0.99)	16.7 (15.1–18.4)	0.57 (0.52–0.61)
Cocktail	0.50 (0.40–0.59)	0.52 (0.41–0.61)	0.97 (0.92–0.99)	24.6 (22.3–27.1)	0.41 (0.37–0.44)

^*a*^ Concordance correlation coefficient (CCC) is the product of the accuracy and precision coefficients where accuracy is used to decide whether there is disagreement between two distributions (mean and variance) of the measures and precision measure variation between two samples. A CCC value of 1 indicates perfect agreement; a value of −1 indicates perfect disagreement, and a value of 0 indicates no agreement. TDI_0.9_ is the difference between matched pairs such that 90% of matched pairs have a difference less than this value. CP_8_ is the proportion of matched pairs with a difference of less than 8.

The cocktail of four phages demonstrated differences in phage activity: testing at site 1 identified eight isolates (PaWRA11285, PaWRA1612, PaWRA2101, PaWRA358800, PaWRA4841, PaWRA552, PaWRA7014, and PaWRA8912) against which the phage cocktail but not any of the individual phages appeared active. Testing at site 2 identified seven isolates (PaWRA097, PaWRA141, PaWRA14981, PaWRA15678, PaWRA16740, PaWRA196, and PaWRA358800) against which the phage cocktail but not any of the individual phages appeared active. The plaque assay identified two isolates (PaWRA14981 and PaWRA15678) against which the phage cocktail but not any of the individual phages appeared active. Conversely, phages found active individually but not in the cocktail included two isolates (PaWRA8914 and PaWRA1612) at site 1, one (PaWRA18560) at site 2, and 10 (PaWRA044, PaWRA100, PaWRA104, PaWRA16344, PaWRA18562, PaWRA25623, PaWRA317, PaWRA358800, PaWRA4841, and PaWRA8141) at site 3.

Results of the subgroup of 46 *P*. *aeruginosa* isolates from cystic fibrosis patients tested at site 1 in triplicate are detailed in Table S1. Phage EPa11 and EPa83 exhibited CCC values over 90% and TDI_0.9_ values below 10. Results of the subgroup of cystic fibrosis isolates tested with two measurements at site 2 are summarized in Table S2 and showed EPa39, EPa87, and the cocktail to have high CCC values and TDI_0.9_ values below 10. Agreement between median site 1 and site 2 results (Table S3) indicated that all statistics except for TDI_0.9_ were lower than the agreement of the measurements within site 1 or 2. Analysis of non-cystic fibrosis isolates (Tables S4 through S6) showed that for phages EPa39 and EPa87 at site 1, the TDI_0.9_ was 3 for phage EPa39 and approximately 10 for phage EPa87; with CP_8_ values of 99% and 85%, respectively. However, for both CF cohort and total isolates (*n* = 145), the TDI_0.9_ was higher, and CP_8_ was lower comparatively. The non-CF cohort accuracy and precision were also higher compared to CF cohort and total isolates (*n* = 145). The reason is likely that the phages were inactive (hold time below 4 hours and inactive per plaque assay) against most of the isolates in the non-CF cohort; hence, the agreement was high. Overall, statistical analysis of the cystic fibrosis subgroup showed results similar to that of the 145 isolates analyzed, with a trend toward site 2 measurements having higher accuracy and agreement than site 1 measurements. A hold time cutpoint of 8 hours gave similar results to a hold time cutpoint of 4 hours (data not shown).

As shown in Table 5, agreement between liquid assays at sites 1 and 2 was 78%, 88%, 76%, 81%, and 77% for phages EPa11, EPa39, EPa83, EPa87, and the cocktail, respectively. When liquid assays were compared to the plaque assay (site 3), however, agreement between the three sites decreased to 63%, 61%, 62%, 62%, and 61% for phages EPa11, EPa39, EPa83, EPa87, and the cocktail, respectively. Overall, there was agreement between all measurements for just 22% of the total 145 bacterial isolates tested.

**TABLE 5:** *Pseudomonas aeruginosa* isolates exhibiting agreement among the measurements of liquid and plaque assay

	Isolate number	Phage EPa11	Phage EPa39	Phage EPa83	Phage EPa87	Phage cocktail
1	PaWRA026	TA^*c*^	NA^*d*^	TA	TA	TA
2	PaWRA03-1	TA	LA^*b*^	TA	NA	TA
3	PaWRA03-2	TA	TA	LA	TA	LA
4	PaWRA030	TA	TA	TA	TA	TA
5	PaWRA032	NA	NA	NA	NA	NA
6	PaWRA042	TA	LA	TA	TA	TA
7	PaWRA043	TA	TA	LA	LA	LA
8	PaWRA044	TA	TA	TA	TA	LA
9	PaWRA045	TA	NA	TA	TA	TA
10	PaWRA046	LA	TA	TA	TA	TA
11	PaWRA047	TA	TA	TA	TA	TA
12	PaWRA048	TA	TA	NA	NA	NA
13	PaWRA059	NA	LA	TA	LA	NA
14	PaWRA064	TA	TA	TA	NA	TA
15	PaWRA067	TA	LA	TA	TA	TA
16	PaWRA069	NA	LA	TA	LA	NA
17	PaWRA071	LA	LA	TA	LA	LA
18	PaWRA081	TA	TA	TA	TA	TA
19	PaWRA083	TA	TA	TA	TA	TA
20	PaWRA087	TA	TA	TA	TA	TA
21	PaWRA093	TA	TA	LA	LA	LA
22	PaWRA095	TA	LA	TA	TA	TA
23	PaWRA096	TA	TA	LA	LA	LA
24	PaWRA097	TA	LA	NA	NA	TA
25	PaWRA098	TA	TA	TA	TA	TA
26	PaWRA099	TA	TA	LA	LA	LA
27	PaWRA100	TA	TA	LA	TA	TA
28	PaWRA104	TA	TA	NA	TA	NA
29	PaWRA11278	TA	TA	TA	NA	TA
30	PaWRA11281	NA	NA	NA	NA	TA
31	PaWRA11285	LA	TA	NA	NA	LA
32	PaWRA11286	TA	TA	TA	TA	TA
33	PaWRA114	LA	LA	TA	TA	TA
34	PaWRA11536	TA	TA	NA	TA	NA
35	PaWRA11538	TA	TA	NA	TA	TA
36	PaWRA11976	LA	LA	TA	TA	LA
37	PaWRA12282	TA	TA	LA	LA	TA
38	PaWRA12283	TA	TA	TA	TA	TA
39	PaWRA12365	TA	TA	TA	TA	TA
40	PaWRA12368	NA	TA	TA	TA	NA
41	PaWRA12914	NA	TA	TA	TA	NA
42	PaWRA130	TA	TA	TA	TA	TA
43	PaWRA132	LA	LA	TA	LA	TA
44	PaWRA1344^*a*^	TA	NA	TA	TA	TA
45	PaWRA13488	TA	TA	TA	TA	TA
46	PaWRA1356^*a*^	TA	TA	TA	TA	TA
47	PaWRA137	TA	TA	TA	TA	TA
48	PaWRA1380^*a*^	TA	TA	LA	LA	LA
49	PaWRA1388^*a*^	TA	TA	TA	TA	TA
50	PaWRA141	TA	TA	NA	TA	TA
51	PaWRA142	TA	TA	TA	LA	TA
52	PaWRA14981	TA	TA	TA	TA	NA
53	PaWRA153	TA	TA	LA	LA	LA
54	PaWRA15566	NA	LA	NA	NA	NA
55	PaWRA15678	NA	TA	TA	TA	TA
56	PaWRA15753	NA	NA	NA	LA	TA
57	PaWRA1583^*a*^	LA	LA	NA	TA	NA
58	PaWRA1601^*a*^	NA	TA	TA	TA	NA
59	PaWRA1612^*a*^	LA	TA	LA	TA	NA
60	PaWRA1613^*a*^	TA	TA	NA	NA	TA
61	PaWRA1617^*a*^	TA	TA	TA	TA	TA
62	PaWRA163	TA	TA	TA	TA	TA
63	PaWRA16344	NA	LA	TA	TA	NA
64	PaWRA16345	TA	TA	NA	NA	TA
65	PaWRA16383	LA	LA	NA	NA	NA
66	PaWRA16740	NA	TA	TA	TA	LA
67	PaWRA16744	NA	LA	TA	TA	NA
68	PaWRA16847	LA	TA	TA	TA	LA
69	PaWRA1688^*a*^	NA	LA	NA	NA	LA
70	PaWRA170	LA	TA	TA	TA	TA
71	PaWRA1739^*a*^	TA	TA	TA	TA	TA
72	PaWRA17849	TA	LA	NA	NA	TA
73	PaWRA183	LA	LA	LA	LA	LA
74	PaWRA18560	TA	NA	NA	TA	NA
75	PaWRA18562	TA	NA	TA	TA	TA
76	PaWRA187	TA	LA	TA	TA	TA
77	PaWRA18754	TA	TA	TA	TA	TA
78	PaWRA18803	LA	LA	TA	TA	LA
79	PaWRA18855	TA	LA	TA	TA	TA
80	PaWRA18970	TA	TA	LA	LA	TA
81	PaWRA1899^*a*^	TA	NA	TA	NA	TA
82	PaWRA1902^*a*^	TA	TA	NA	NA	NA
83	PaWRA1906^*a*^	TA	TA	NA	TA	NA
84	PaWRA1925^*a*^	LA	TA	TA	LA	TA
85	PaWRA1938^*a*^	TA	TA	LA	LA	LA
86	PaWRA1948^*a*^	NA	LA	NA	TA	NA
87	PaWRA196	TA	TA	TA	TA	NA
88	PaWRA19711	LA	NA	TA	TA	NA
89	PaWRA20176	TA	TA	NA	NA	NA
90	PaWRA20190	TA	TA	NA	NA	TA
91	PaWRA2101^*a*^	NA	LA	TA	TA	TA
92	PaWRA2108^*a*^	NA	LA	TA	TA	NA
93	PaWRA2144^*a*^	LA	LA	TA	TA	TA
94	PaWRA23861	LA	LA	TA	LA	TA
95	PaWRA2444^*a*^	TA	TA	TA	TA	TA
96	PaWRA25623	TA	TA	TA	NA	LA
97	PaWRA25678	TA	TA	TA	TA	TA
98	PaWRA25762	TA	TA	TA	TA	TA
99	PaWRA26263	TA	TA	TA	TA	NA
100	PaWRA29192	TA	NA	LA	TA	TA
101	PaWRA300	TA	TA	NA	LA	TA
102	PaWRA301	TA	TA	TA	TA	TA
103	PaWRA302	TA	TA	TA	TA	TA
104	PaWRA304	TA	TA	TA	LA	TA
105	PaWRA305	TA	TA	NA	NA	NA
106	PaWRA306	TA	LA	TA	NA	NA
107	PaWRA30858	TA	NA	LA	LA	TA
108	PaWRA315^*a*^	NA	TA	NA	NA	TA
109	PaWRA317^*a*^	TA	LA	TA	TA	TA
110	PaWRA321^*a*^	TA	TA	NA	TA	TA
111	PaWRA346179	NA	NA	TA	TA	NA
112	PaWRA351791	NA	LA	TA	NA	TA
113	PaWRA358800	NA	LA	TA	TA	LA
114	PaWRA369569	TA	TA	TA	TA	TA
115	PaWRA3705^*a*^	TA	TA	TA	TA	TA
116	PaWRA373401	TA	TA	TA	TA	TA
117	PaWRA390231	TA	LA	TA	TA	TA
118	PaWRA401528	LA	TA	TA	TA	TA
119	PaWRA409937	LA	LA	TA	TA	NA
120	PaWRA435288	NA	TA	TA	TA	TA
121	PaWRA436311	LA	LA	LA	TA	TA
122	PaWRA443463	LA	LA	TA	NA	TA
123	PaWRA4841^*a*^	NA	LA	TA	TA	LA
124	PaWRA5498^*a*^	NA	TA	TA	TA	NA
125	PaWRA5508^*a*^	TA	TA	NA	TA	TA
126	PaWRA5519^*a*^	NA	LA	NA	LA	TA
127	PaWRA552^*a*^	NA	LA	NA	TA	TA
128	PaWRA5524^*a*^	TA	TA	TA	TA	TA
129	PaWRA5539^*a*^	TA	LA	TA	LA	TA
130	PaWRA6220^*a*^	TA	TA	TA	TA	TA
131	PaWRA6241^*a*^	TA	TA	TA	TA	TA
132	PaWRA6678^*a*^	TA	TA	NA	NA	NA
133	PaWRA6695^*a*^	NA	NA	NA	NA	NA
134	PaWRA6739^*a*^	TA	LA	LA	LA	TA
135	PaWRA7014^*a*^	TA	TA	NA	NA	TA
136	PaWRA8130^*a*^	NA	LA	LA	TA	TA
137	PaWRA8136^*a*^	TA	TA	TA	TA	TA
138	PaWRA8139^*a*^	NA	NA	NA	LA	LA
139	PaWRA8141^*a*^	LA	TA	TA	TA	TA
140	PaWRA8912^*a*^	NA	TA	TA	TA	LA
141	PaWRA8914^*a*^	NA	TA	TA	TA	TA
142	PaWRA8915^*a*^	TA	TA	TA	TA	TA
143	PaWRA9718^*a*^	TA	TA	LA	LA	LA
144	PaWRA9873^*a*^	TA	TA	TA	TA	TA
145	PaWRA994^*a*^	TA	TA	LA	LA	LA
Isolates with liquid assay agreement		114	129	111	118	113
% isolates in liquid assay agreement		79	89	77	81	78
**I**solates with total agreement		92	89	91	91	89
% isolates in total agreement		63	61	63	63	61

^*a*^ Isolates from cystic fibrosis patients.

^*b*^ LA, liquid assay agreement.

^*c*^ TA, total agreement.

^*d*^ NA, no liquid assay agreement.

**Fig 3:**
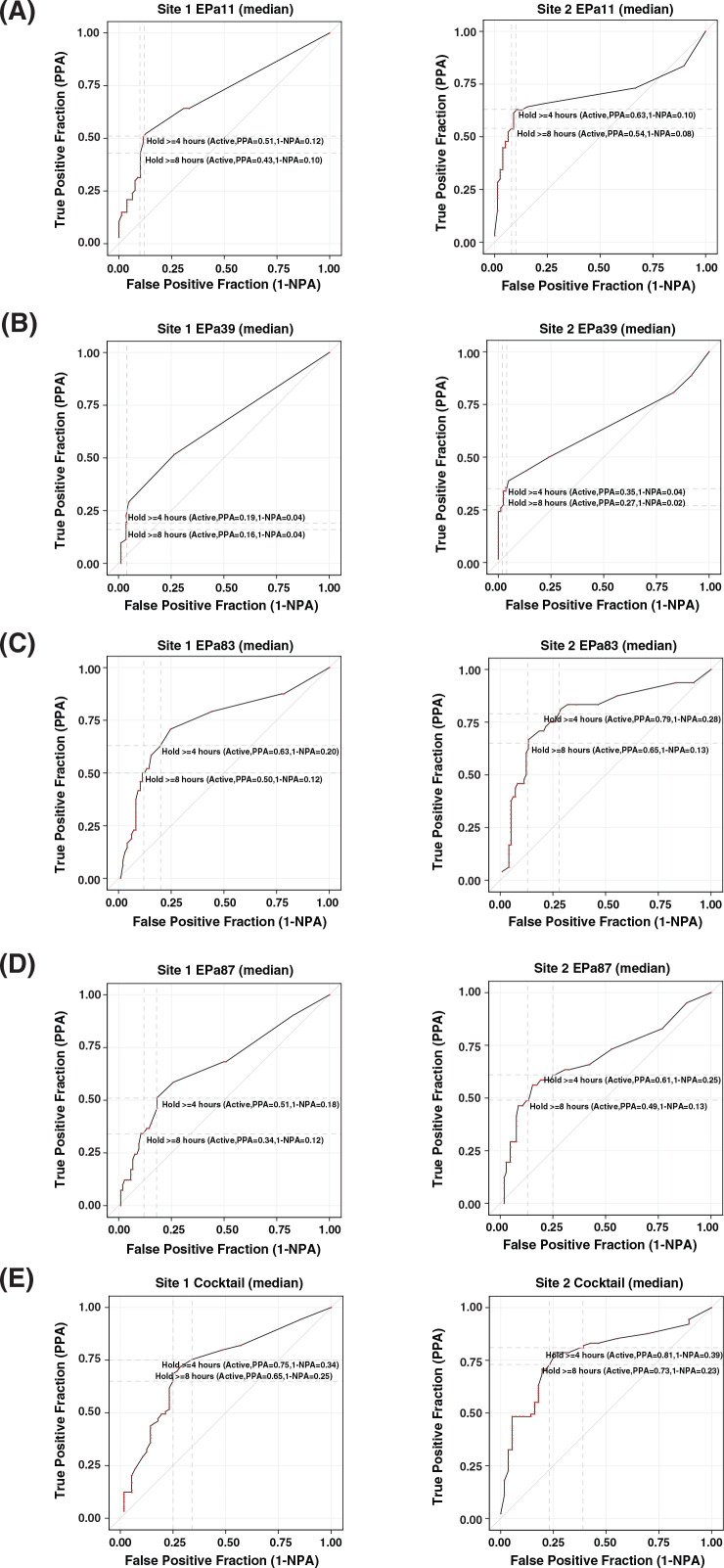
Positive percent agreement (PPA)/negative percent agreement (NPA) plot comparing site 1 (left) and 2 (right) liquid assay testing to site 3 plaque assay testing for (**A**) phage EPa11, (**B**) phage EPA39, (**C**) phage EPa83, (**D**) phage EPa87, and (**E**) phage cocktail. The true positive fraction/false positive fraction is marked at liquid assay cutpoints of 4 and 8 hours.

Fig. 3 illustrates PPA/NPA plots of both laboratory liquid assay measurements as their discrimination threshold varies for the five phage preparations (four individual phages and the cocktail). The PPA/NPA plots are shown with the hold times of ≥4 and ≥8 hours used as cutpoints for active phages. For phages EPa11 and EPa39, site 2 showed higher true positive fraction (sensitivity) at both cutpoints compared to site 1. The cutpoint of ≥4 hours was associated with a higher true positivity/sensitivity than the cutpoint of ≥8 hours. For phage EPa83 and EPa87, the true positive fraction/sensitivity at both cutpoints was higher, while the false positive fraction (1 − NPA) was slightly lower for site 2 as compared with site 1. For the phage cocktail, at both cutpoints, site 2 showed higher true positivity/sensitivity and comparable false positivity to site 1. Also, among all phage preparations, the cocktail had the highest true positivity for both sites.

## DISCUSSION

This study reports a comparison of a liquid assay with a plaque assay method, including within- and between-laboratory agreements for the former. For the liquid assay, there was high agreement between duplicate measurements made on the same run at site 2 and satisfactory agreement among triplicate measurements made on different days at site 1. Triplicate measurements were performed on different days, a more rigorous assessment than the duplicate measurements which were performed on the same run. By testing on different days, issues such as phage storage and fresh preparation of medium with tetrazolium dye, and phage and bacterial inocula may affect results. There was lack of agreement between results from the two sites performing the liquid assay, with CCCs below 0.6 and low precision for all phages tested. Other investigators have reported inconsistencies among measurements when performing *P. aeruginosa* PST (16 – 18). The liquid assay method used in this study did not use optical densitymeasurements but conversion of a water-soluble tetrazolium salt, which potentially yields a higher sensitivity and dynamic range. Among 725 points of comparison (5 phage preparations and 145 bacterial isolates), 22% (32 of 145) of bacterial isolates showed agreement between all measurements, whether by liquid assay at two sites or plaque assay, with 61% (89 of 145) yielding agreement between the liquid assay at minimally one site and the plaque assay. Liquid assays may yield falsely negative results if phage resistance is acquired early during the assay. Interesting differences between activity of individual phages and phages in the cocktail preparation were observed for a small number of phages; these differences were not consistent between liquid and plaque assays. Synergistic, neutral, and antagonistic behaviors of phages in a cocktail have been reported to be independent of the behavior of individual phages even at the same MOIs, incubation temperatures, exposure times, and bacterial strains, and are influenced by phage genera and phage-phage interactions (19). The activity of a few individual phages assessed as inactive (i.e., below a cutpoint of 4 hours) seemed to be enhanced when the phages were assessed as a cocktail, making the cocktail a treatment that deserves further study. It is possible that the type and characteristics of each individual phage and/or bacterial host may be unique and that these uniquely impact methodological reproducibility. Phage infection depends on host bacterial physiology; given that bacterial growth can depend on the nutritional media used, dynamics in liquid and solid media may be distinct and vary from isolate to isolate (20), based on differences in adsorption rate, burst size, and/or latent period (21).

When disagreements were found between methods, the plaque assay tended to indicate higher levels of phage activity. Of the 725 phage-bacteria interactions, 307 (42%) showed activity as assessed by the plaque assay in comparison to 285 (39%) by the liquid assay performed at site 2 and 236 (32%) at site 1. When the liquid assay resulted in inactive phage (i.e., below a 4-hour cutpoint), there were only 8% of the isolates for which the hold times were relatively close to 4 hours (i.e., 3.1–3.8 hours) with the remaining being below 3 hours. Plaque assays may indicate a clear plaque, whereas the amount of time to regrowth of bacteria is monitored via a liquid-based assay. Plaque assays are generally qualitative tests, whereas there is an absolute value of hold time observed via liquid assays. The potential clinical implications of the plaque assay tending to indicate higher levels of plaque activity are unknown. The spatial dynamics of soft agar may play a role in phage diffusion and thereby formation of plaques. In semisolid media, phage diffusion, mobility, and mixing may be slowed compared to liquid media, such that absence of signal may not always indicate an inability of the phage to infect the bacterial strain, although this is unlikely to explain the observed increased level of activity with the plaque assay in this study. Another challenge with plaque assays is variability in observer readings. In a study by Henry et al. (22), soft agar layer spot assays overestimated the EOP of phage PhiKZ such that in liquid culture, results were contrary. Another study using *Salmonella* phage P16 reported different results between a soft agar spot method and a liquid culture assay (23). There are also limitations to study design where triplicate measurements for site 1 were performed on different days; duplicate measurements for site 2 were performed on the same day; and the plaque assay for site 3 was performed only once, which hamper making conclusions regarding the liquid versus plaque comparison and between the two sites performing liquid assays. A final limitation is the poor overall coverage of the phages studied.

Prior studies have highlighted gaps in agreement of PST assays and approaches (24), with specificity and promiscuity of phages toward bacterial hosts varying among different testing methods (25 – 27). Further studies of phage biology and phage interactions with bacterial hosts may help determine which PST methods are ideal (28 – 30). Assay reproducibility is a critical step in the development of PST; ultimately, results of PST require correlation with clinical outcomes from rigorous clinical trials. While susceptibility testing for conventional antibiotics has been standardized over decades, it has known complexity and variability. PST variability adds to that of standard antibiotic testing, with the additional biological complexities of phages; that is, the action of a virus that infects a bacterium in a multi-step biological process to kill it versus the action of a single-molecule drug such as an antimicrobial agent (i.e., chemical).

In conclusion, to date, no standardized PST method exists. Results of this study suggest that PST needs further development to deliver reproducible methods. Replicate testing and rigorous wet laboratory method standardization are needed to normalize assays and minimize variability while considering the importance of assessing PST in support of clinical trials of phage therapy.

## SUPPLEMENTAL MATERIAL

Supplemental file 1 jcm.00614-23-s0001.tif Fig. S1.

Supplemental file 2 jcm.00614-23-s0002.tif Fig. S2.

Supplemental file 3 jcm.00614-23-s0003.docx Supplemental figure legends.

Supplemental file 4 jcm.00614-23-s0004.docx Tables S1 to S6.

ASM does not own the copyrights to Supplemental Material that may be linked to, or accessed through, an article. The authors have granted ASM a non-exclusive, world-wide license to publish the Supplemental Material files. Please contact the corresponding author directly for reuse.
