# Supplementary figures and images for: Interlaboratory comparison of Pseudomonas aeruginosa phage susceptibility testing
Source: J Clin Microbiol. 2023 Nov 14;61(12):e00614-23. doi: 10.1128/jcm.00614-23 (PMC10729752; doi:10.1128/jcm.00614-23)

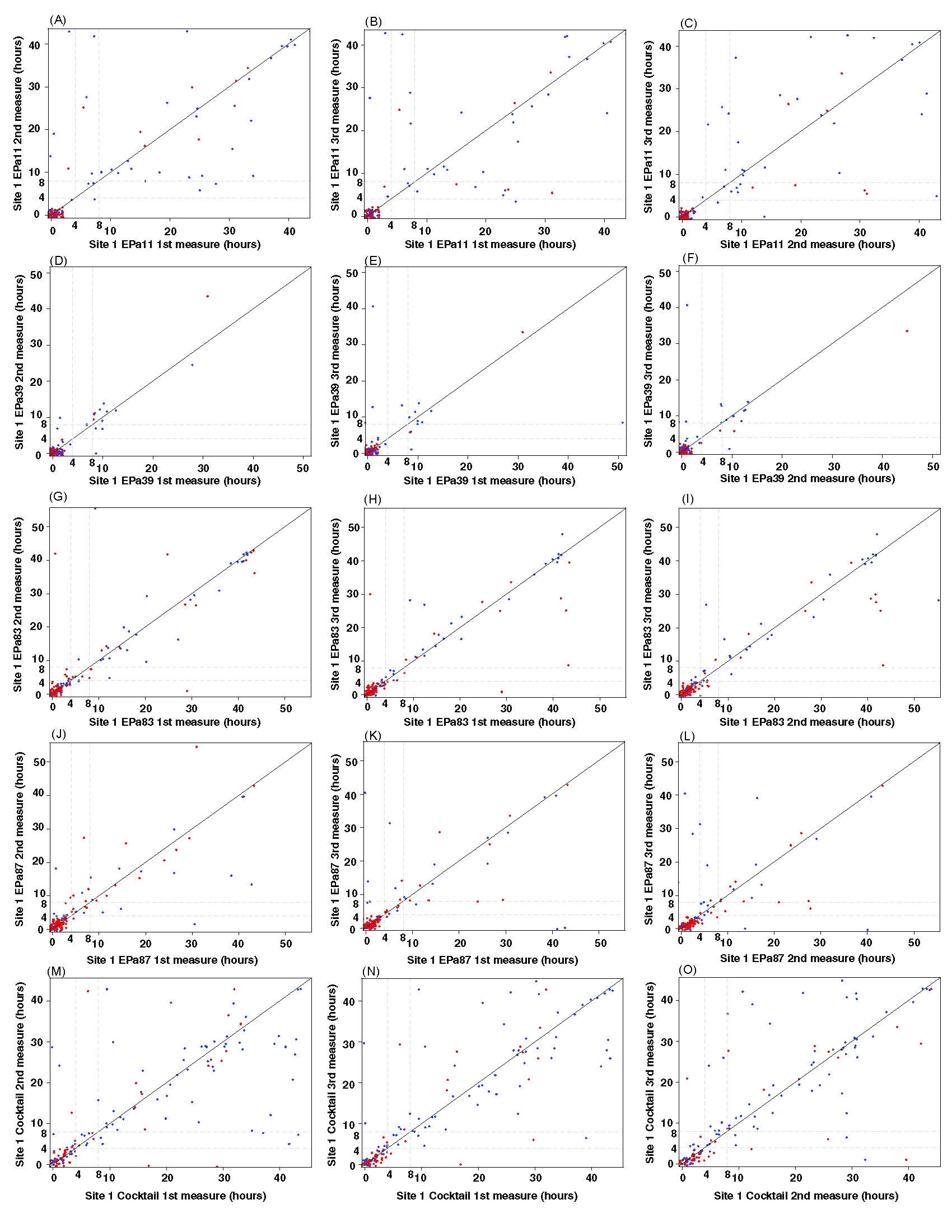

Supplement: Supplemental file 1 — Fig. S1. [file jcm.00614-23-s0001.tif]

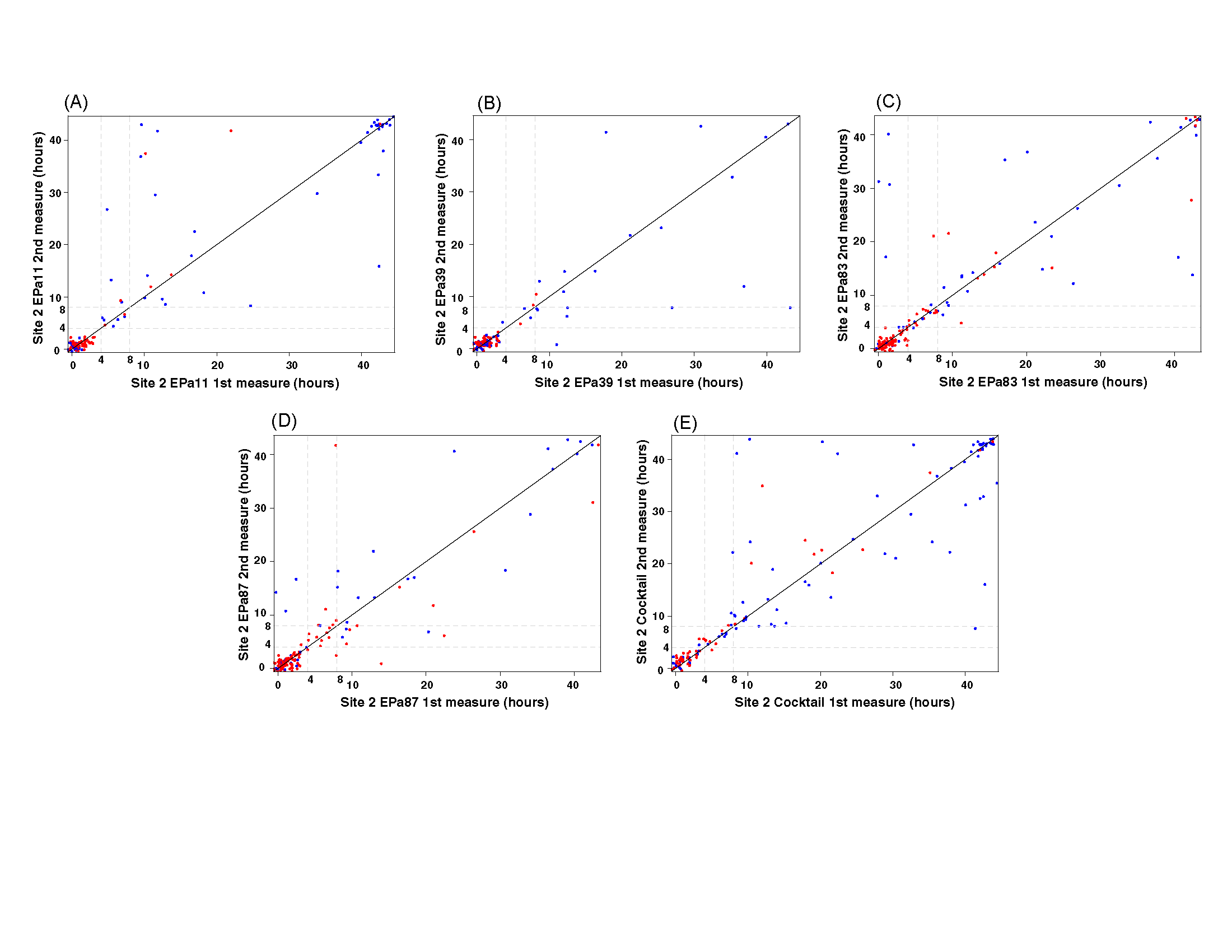

Supplement: Supplemental file 2 — Fig. S2. [file jcm.00614-23-s0002.tif]
