# Supplementary material for: Interlaboratory comparison of Pseudomonas aeruginosa phage susceptibility testing
Source: J Clin Microbiol. 2023 Nov 14;61(12):e00614-23. doi: 10.1128/jcm.00614-23 (PMC10729752; doi:10.1128/jcm.00614-23)
Supplement: Supplemental file 3 — Supplemental figure legends. [file jcm.00614-23-s0003.docx]

**Supplementary Figure 1:** Scatterplot comparing agreement among three measurements at site 1. Comparisons are shown are between first-second, second-third, and first-third measurements at site 1, using liquid assay breakpoints of four and eight hours. Color coding is based on plaque assays at site 3: red - inactive, blue - active. (A) phage EPa11 – comparison between first and second measurement, (B) phage EPa11 – comparison between first and third measurement, (C) phage EPa11 – comparison between second and third measurement, (D) phage EPa39 – comparison between first and second measurement, (E) phage EPa39 – comparison between first and third measurement, (F) phage EPa39 – comparison between second and third measurement, (G) phage EPa83 – comparison between first and second measurement, (H) phage EPa83 – comparison between first and third measurement, (I) phage EPa83 – comparison between second and third measurement, (J) phage EPa87 – comparison between first and second measurement, (K) phage EPa87 – comparison between first and third measurement, (L) phage cocktail – comparison between second and third measurement, (M) phage cocktail – comparison between first and second measurement, (N) phage cocktail – comparison between first and third measurement, and (O) phage cocktail – comparison between second and third measurement.

**Supplementary Figure 2:** Scatterplot comparing agreement among values of two measurements at site 2. Comparisons are between first and second measurements at site 2, using liquid assay breakpoints of four and eight hours. Color coding is based on plaque assays at site 3: red - inactive, blue - active. (A) phage EPa11 – comparison between first and second measurement, (B) phage EPa39 – comparison between first and second measurement, (C) phage EPa83 – comparison between first and second measurement, (D) phage EPa87 – comparison between first and second measurement, and (E) phage cocktail – comparison between first and second measurement.
