# Supplementary material for: Interlaboratory comparison of Pseudomonas aeruginosa phage susceptibility testing
Source: J Clin Microbiol. 2023 Nov 14;61(12):e00614-23. doi: 10.1128/jcm.00614-23 (PMC10729752; doi:10.1128/jcm.00614-23)
Supplement: Supplemental file 4 — Tables S1 to S6. [file jcm.00614-23-s0004.docx]

**Supplementary Table 1: Agreement between maximum and minimum of three measurements at site 1 in the cystic fibrosis cohort (N=46, TDI_0.9_, CP_8_)**

| **Phage** | **Concordance Correlation Coefficient (95% CI)** | **Precision (95% CI)** | **Accuracy (95% CI)** | **TDI_0.9_ (95% CI)** | **CP_8_ (95% CI)** |
| --- | --- | --- | --- | --- | --- |
| EPa11 | 0.91 (0.87-0.93) | 0.93 (0.89-0.95) | 0.98 (0.95-0.99) | 9.48 (8.03-11.2) | 0.83 (0.75-0.89) |
| EPa39 | 0.45 (0.31-0.57) | 0.61 (0.45-0.73) | 0.74 (0.65-0.81) | 16.4 (14.6-18.3) | 0.82 (0.77-0.87) |
| EPa83 | 0.97 (0.96-0.98) | 0.98 (0.98-0.99) | 0.99 (0.98-0.99) | 5.18 (4.44-6.06) | 0.991 (0.97-0.99) |
| EPa87 | 0.51 (0.39-0.62) | 0.67 (0.53-0.78) | 0.76 (0.67-0.83) | 20.0 (17.7-22.5) | 0.61 (0.55-0.66) |
| Cocktail | 0.70 (0.61-0.78) | 0.81 (0.73-0.87) | 0.87 (0.80-0.92) | 17.6 (15.3-20.2) | 0.55 (0.49-0.61) |

**Concordance Correlation Coefficient (CCC)** is the product of the accuracy and precision coefficients where **accuracy** is used to decide whether there is disagreement between two distributions (mean and variance) of the measures and **precision** measures variation between two samples. A CCC value of 1 indicates perfect agreement, a value of -1 indicates perfect disagreement, and a value of 0 indicates no agreement. **TDI_0.9_** is the difference between matched pairs such that 90% of matched pairs have a difference less than this value. **CP_8_** is the proportion of matched pairs with a difference less than 8.

**Supplementary Table 2: Agreement between duplicate measurements at site 2 in the cystic fibrosis cohort (N=46, TDI_0.9_, CP_8_)**

| **Phage** | **Concordance Correlation Coefficient (95% CI)** | **Precision (95% CI)** | **Accuracy (95% CI)** | **TDI_0.9_ (95% CI)** | **CP_8_ (95% CI)** |
| --- | --- | --- | --- | --- | --- |
| EPa11 | 0.81 (0.72-0.87) | 0.85 (0.76-0.91) | 0.95 (0.89-0.98) | 10.4 (8.72-12.4) | 0.79 (0.69-0.86) |
| EPa39 | 0.89 (0.83-0.93) | 0.91 (0.86-0.95) | 0.97 (0.93-0.99) | 5.93 (4.97-7.06) | 0.97 (0.92-0.99) |
| EPa83 | 0.73 (0.59-0.82) | 0.74 (0.60-0.83) | 0.98 (0.89-0.99) | 16.9 (14.2-20.2) | 0.55 (0.47-0.63) |
| EPa87 | 0.92 (0.88-0.95) | 0.93 (0.89-0.96) | 0.99 (0.96-0.99) | 8.13 (6.83-9.69) | 0.89 (0.81-0.94) |
| Cocktail | 0.92 (0.88-0.95) | 0.92 (0.88-0.95) | 0.99 (0.96-1.00) | 9.82 (8.24-11.7) | 0.81 (0.72-0.88) |

**Concordance Correlation Coefficient (CCC)** is the product of the accuracy and precision coefficients where **accuracy** is used to decide whether there is disagreement between two distributions (mean and variance) of the measures and **precision** measures variation between two samples. A CCC value of 1 indicates perfect agreement, a value of -1 indicates perfect disagreement, and a value of 0 indicates no agreement. **TDI_0.9_** is the difference between matched pairs such that 90% of matched pairs have a difference less than this value. **CP_8_** is the proportion of matched pairs with a difference less than 8.

**Supplementary Table 3: Agreement between median measurements at sites 1 and 2 in the cystic fibrosis cohort (N=46, TDI_0.9_, CP_8_)**

| **Phage** | **Concordance Correlation Coefficient (95% CI)** | **Precision (95% CI)** | **Accuracy (95% CI)** | **TDI_0.9_ (95% CI)** | **CP_8_ (95% CI)** |
| --- | --- | --- | --- | --- | --- |
| EPa11 | 0.64 (0.49-0.75) | 0.69 (0.54-0.80) | 0.93 (0.83-0.97) | 16. 5 (13.8-19.6) | 0.57 (0.48-0.65) |
| EPa39 | 0.51 (0.31-0.66) | 0.52 (0.31-0.68) | 0.98 (0.84-0.997) | 11.2 (9.43-13.4) | 0.75 (0.66-0.82) |
| EPa83 | 0.71 (0.57-0.81) | 0.71 (0.56-0.82) | 0.999 (0.44-1.00) | 16. 4 (13.8-19.5) | 0.57 (0.49-0.65) |
| EPa87 | 0.42 (0.20-0.60) | 0.43 (0.20-0.61) | 0.995 (0.61-1.00) | 20.8 (17.5-24. 8) | 0.46 (0.39-0.54) |
| Cocktail | 0.67 (0.51-0.79) | 0.67 (0.51-0.79) | 0.997 (0.59-1.00) | 19.7 (16.5-23. 5) | 0.49 (0.41-0.56) |

**Concordance Correlation Coefficient (CCC)** is the product of the accuracy and precision coefficients where **accuracy** is used to decide whether there is disagreement between two distributions (mean and variance) of the measures and **precision** measures variation between two samples. A CCC value of 1 indicates perfect agreement, a value of -1 indicates perfect disagreement, and a value of 0 indicates no agreement. **TDI_0.9_** is the difference between matched pairs such that 90% of matched pairs have a difference less than this value. **CP_8_** is the proportion of matched pairs with a difference less than 8.

**Supplementary Table 4: Agreement between maximum and minimum of three measurements at site 1 in the non-cystic fibrosis cohort (N=99, TDI_0.9_, CP_8_)**

| **Phage** | **Concordance Correlation Coefficient (95% CI)** | **Precision (95% CI)** | **Accuracy (95% CI)** | **TDI_0.9_ (95% CI)** | **CP_8_ (95% CI)** |
| --- | --- | --- | --- | --- | --- |
| EPa11 | 0.51 (0.44-0.58) | 0.70 (0.62-0.77) | 0.73 (0.67-0.78) | 19.06 (17.73-20.48) | 0.65 (0.72-0.68) |
| EPa39 | 0.76 (0.71-0.81) | 0.85 (0.81-0.88) | 0.89 (0.85-0.92) | 3.65 (3.32-4.00) | 0.99 (0.99-1.00) |
| EPa83 | 0.74 (0.67-0.79) | 0.82 (0.77-0.86) | 0.90 (0.86-0.93) | 14.10 (12.84-15.50) | 0.73 (0.69-0.77) |
| EPa87 | 0.69 (0.62-0.74) | 0.78 (0.72-0.83) | 0.88 (0.83-0.91) | 10.00 (9.11-10.98) | 0.85 (0.83-0.88) |
| Cocktail | 0.68 (0.62-0.74) | 0.80 (0.74-0.84) | 0.86 (0.81-0.89) | 18.16 (16.57-19.91) | 0.54 (0.50-0.58) |

**Concordance Correlation Coefficient (CCC)** is the product of the accuracy and precision coefficients where **accuracy** is used to decide whether there is disagreement between two distributions (mean and variance) of the measures and **precision** measures variation between two samples. A CCC value of 1 indicates perfect agreement, a value of -1 indicates perfect disagreement, and a value of 0 indicates no agreement. **TDI_0.9_** is the difference between matched pairs such that 90% of matched pairs have a difference less than this value. **CP_8_** is the proportion of matched pairs with a difference less than 8.

**Supplementary Table 5: Agreement between maximum and minimum of two measurements at site 2 in the non-cystic fibrosis cohort (N=99, TDI_0.9_, CP_8_)**

| **Phage** | **Concordance Correlation Coefficient (95% CI)** | **Precision (95% CI)** | **Accuracy (95% CI)** | **TDI_0.9_ (95% CI)** | **CP_8_ (95% CI)** |
| --- | --- | --- | --- | --- | --- |
| EPa11 | 0.92 (0.89-0.94) | 0.92 (0.89-0.94) | 0.99 (0.98-1.00) | 10.89 (9.68-12.26) | 0.76 (0.70-0.81) |
| EPa39 | 0.81 (0.75-0.86) | 0.83 (0.77-0.88) | 0.97 (0.94-0.99) | 8.45 (7.51-9.51) | 0.87 (0.82-0.91) |
| EPa83 | 0.94 (0.91-0.95) | 0.94 (0.92-0.95) | 0.99 (0.98-0.99) | 6.97 (6.20-7.85) | 0.93 (0.89-0.96) |
| EPa87 | 0.88 (0.84-0.91) | 0.88 (0.84-0.91) | 0.99 (0.96-1.00) | 8.01 (7.12-9.01) | 0.89 (0.84-0.93) |
| Cocktail | 0.90 (0.86-0.92) | 0.90 (0.86-0.92) | 1.00 (0.91-1.00) | 12.61 (11.20-14.19) | 0.69 (0.63-0.75) |

**Concordance Correlation Coefficient (CCC)** is the product of the accuracy and precision coefficients where **accuracy** is used to decide whether there is disagreement between two distributions (mean and variance) of the measures and **precision** measures variation between two samples. A CCC value of 1 indicates perfect agreement, a value of -1 indicates perfect disagreement, and a value of 0 indicates no agreement. **TDI_0.9_** is the difference between matched pairs such that 90% of matched pairs have a difference less than this value. **CP_8_** is the proportion of matched pairs with a difference less than 8.

**Supplementary Table 6: Agreement between median measurements at sites 1 and 2 in the non-cystic fibrosis cohort (N=99, TDI_0.9_, CP_8_)**

| **Phage** | **Concordance Correlation Coefficient (95% CI)** | **Precision (95% CI)** | **Accuracy (95% CI)** | **TDI_0.9_ (95% CI)** | **CP_8_ (95% CI)** |
| --- | --- | --- | --- | --- | --- |
| EPa11 | 0.37 (0.24-0.49) | 0.43 (0.28-0.55) | 0.88 (0.79-0.93) | 26.64 (23.68-29.96) | 0.37 (0.33-0.41) |
| EPa39 | 0.40 (0.33-0.47) | 0.67 (0.57-0.75) | 0.60 (0.53-0.66) | 11.52 (10.25-12.94) | 0.74 (0.68-0.79) |
| EPa83 | 0.46 (0.32-0.58) | 0.47 (0.33-0.59) | 0.99 (0.90-0.99) | 20.16 (17.92-22.69) | 0.48 (0.43-0.53) |
| EPa87 | 0.48 (0.35-0.59) | 0.51 (0.38-0.63) | 0.93 (0.86-0.97) | 14.51 (12.89-16.32) | 0.63 (0.57-0.68) |
| Cocktail | 0.42 (0.29-0.54) | 0.45 (0.31-0.57) | 0.94 (0.86-0.97) | 26.60 (23.64-29.93) | 0.37 (0.33-0.41) |

**Concordance Correlation Coefficient (CCC)** is the product of the accuracy and precision coefficients where **accuracy** is used to decide whether there is disagreement between two distributions (mean and variance) of the measures and **precision** measures variation between two samples. A CCC value of 1 indicates perfect agreement, a value of -1 indicates perfect disagreement, and a value of 0 indicates no agreement. **TDI_0.9_** is the difference between matched pairs such that 90% of matched pairs have a difference less than this value. **CP_8_** is the proportion of matched pairs with a difference less than 8.
